# Where to Make Hypodermic Injections

**Published:** 1876-05

**Authors:** 


Where to Make Hypodermic Injections.—The point of puncture is of greater importance than is generally conceded or thought of, so far as making the operation painless and leaving no painful after effects. Never insert *below* the elbow, for obvious reasons: it is far more painful and the irritation greater and of longer duration. At or near the insertion of the deltoid muscle is the *best* place; and next, in front, between the ribs and point of the hip bone; from near the spine to the median line, if the patient is at all fleshy. At these points, if skillfully introduced, it is almost without pain, and there is but little subsequent irritation or continued tenderness.—*Greene: Richmond and Louisville Journal*, January, 1876.

